# Effects of *CYP2B6* polymorphisms on plasma nevirapine concentrations: a systematic review and meta-analysis

**DOI:** 10.1038/s41598-020-74506-x

**Published:** 2020-10-15

**Authors:** Ha Young Yoon, Young Ah Cho, Jeong Yee, Hye Sun Gwak

**Affiliations:** 1 College of Pharmacy and Graduate School of Pharmaceutical Sciences, Ewha Womans University, 52 Ewhayeodae-gil, Seodaemun-gu, Seoul, 03760 Republic of Korea; 2 College of Pharmacy, Gyeongsang National University, Jinju, Gyeongnam 52828 Republic of Korea; 3 Mokhwa Convalescent Hospital, Jinju, Gyeongnam 52828 Republic of Korea

**Keywords:** Genetics, Molecular biology

## Abstract

Cytochrome P450 (CYP) is involved in the metabolism of nevirapine (NVP); especially, CYP2B6 has been known to be one of the main enzymes involved in NVP metabolism. The objective of this study was to investigate the effects of *CYP2B6* variants on plasma concentrations of NVP by a systematic review and meta-analysis. A search for qualifying studies published until April 2020 was conducted using the EMBASE, PubMed, and Web of Science databases. The mean difference (MD) and 95% confidence intervals (CIs) were calculated. Data analysis was performed using R Studio (version 3.6) and Review Manager (version 5.3). In total, data from six studies involving 634 patients were analyzed in the systematic review and five studies in the meta-analysis. We found that carriers of the *CYP2B6* 516TT genotype had a 2.18 µg/mL higher NVP concentration than did the GG or GT (95% CI 1.28–3.08). In the respective comparisons of the three genotypes, it was found that the MD was 1.87 µg/mL between the TT and GT groups, 2.53 µg/mL between TT and GG, and 0.60 µg/mL between GT and GG. This meta-analysis confirmed that *CYP2B6* polymorphisms was associated with plasma NVP concentrations. Therefore, *CYP2B6* genotyping may be useful to predict the responses to NVP.

## Introduction

Acquired Immunodeficiency Syndrome (AIDS) is one of the greatest public health challenges, with a World Health Organization (WHO) estimate of 37.9 million infected people around the world^1^. Ever since highly active antiretroviral therapy (HAART) was introduced in 1996, AIDS prognosis has greatly improved^2^. Typically, HAART targets multiple viral replication cycles and includes two nucleoside reverse transcriptase inhibitors (NRTIs), a non-nucleoside reverse transcriptase inhibitor (NNRTI), and a protease inhibitor (PI) or an integrase inhibitor (INSTI)^3^. Especially, NNRTIs decrease HIV-1 reverse transcriptase activity through allosteric inhibition.

Nevirapine (NVP), due to its low cost and high efficacy, is one of the most frequently utilized NNRTIs^4,5^. In NVP treatment, a significant relationship between trough concentrations and virologic response or toxicity has been reported^6^. Meanwhile, inter-individual variability in NVP plasma concentrations was observed to be approximately 50%, where such variability could partially be explained by differences in ethnicity, gender, existence of hepatic disease and concomitant medications^7–9^. Additionally, genetic disposition was also shown to contribute to inter-individual variability in NVP concentrations^10^. Among the various genetic factors that may be associated with individual variability in NVP concentrations, genetic polymorphisms of the enzyme cytochrome P450 (CYP) are prime candidates, as NVP is principally metabolized by CYP3A4 and CYP2B6 into its major metabolites^11,12^.

NVP undergoes significant oxidative metabolism to 2-, 3-, 8- and 12-hydroxynevirapine and 4-carboxynevirapine, followed by glucuronidation and renal excretion. A study reported that more than 80% of the radioactivity in urine was consisted of glucuronidated conjugates of hydroxylated NVP metabolites^12^. Among the metabolites, 2- and 3-hydroxynevirapine are known to be formed exclusively by CYP3A4 and CYP2B6, respectively. Since NVP has been suggested to be an inducer of the CYPs responsible for its own metabolism, and hepatic CYP2B genes are considered as the most-inducible CYP isoforms^13^, CYP2B6 could play an important role in NVP metabolism.

Several studies have investigated the association between *CYP2B6* gene polymorphisms and NVP concentration. However, since most such studies did not utilize a substantially large sample size, broad conclusions from separate studies are not readily attainable. Instead, assimilating data from such studies and deducing meaningful results from the pile of information is both prudent and necessary. In this context, we performed a meta-analysis of the published literature in search of significant conclusions on the effects of *CYP2B6* genetic variants on plasma NVP concentrations.

## Results

### Literature search

**Figure 1:**
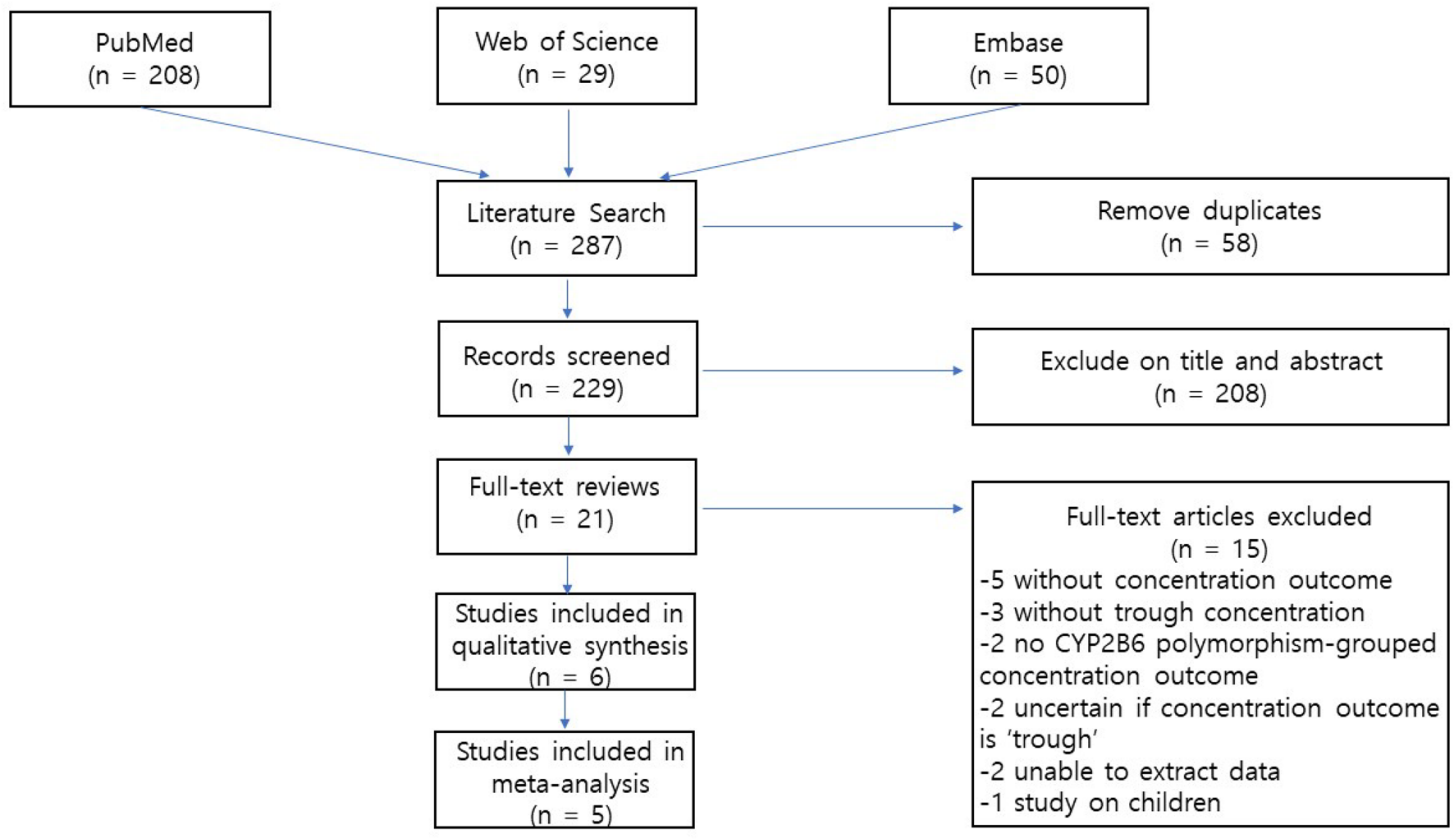
Flow diagram of included studies.

A detailed flow chart of the study selection process is presented in Fig. 1. A total of 287 studies were identified from searches of three databases. After removal of 58 duplicates, 229 records were initially identified, of which the titles and abstracts were screened for inclusion in the study. From this initial review, 21 studies were selected for full-text reviews and assessed for eligibility. Of these studies, 15 were excluded for the following reasons; there were no concentration outcomes (n = 5), no trough concentration (n = 3), no *CYP2B6* polymorphism-grouped concentration outcomes (n = 2), the researchers were not sure if the concentration outcomes were trough (n = 2), unable to extract the data (*n* = 2) and a study on children (n = 1). Although the study by Manhungu^14^ had one patient who received 600 mg once daily, we included the study because most patients (99%) underwent either 400 mg once daily or 200 mg twice daily. Ultimately, six articles were selected for this systematic review^14–19^.

The characteristics of the included studies are presented in Table 1. The studies were published from 2009 to 2018. Two of them were conducted in Asia, one in Africa, and three in Europe. There were three randomized controlled trials, two cohort studies, and one cross-sectional study, with Joanna Briggs Institutes (JBI) Critical Appraisal scores^20^ ranging from 6 to 9.

**Table 1:** Characteristics of studies included in the systematic review.

First author	Study design	Sample size (male %)	Age (years) (mean ± SD)	Country	Alleles studied	Quality score
Mahungu, 2009	Cohort	104 (82.0)	44.5 ± 6.7	UK	516 G > T, 983 T > C, 1459 C > T	7/11
Uttayamakul, 2010	RCT	59 (68.0)	38.0 ± 8.6	Thailand	516 G > T	6/13
Gozalo, 2011	RCT	72 (74.0)	35.7 ± 6.8	France	516 G > T, 785 A > G, 1459 C > T	6/13
Calcagno, 2012	Cross-sectional	204 (29.9)	39.3 ± 11.2	Burundi	516 G > T, 983 T > C	6/8
Ramachandran, 2013	RCT	52 (80.8)	38.0 ± 7.9	India	516 G > T	6/13
Giacomelli, 2018	Cohort	143 (61.5)	47.7 ± 7.7	Italy	516 G > T	9/11

*RCT* randomized controlled trial.

### CYP2B6 516 G > T

**Figure 2:**
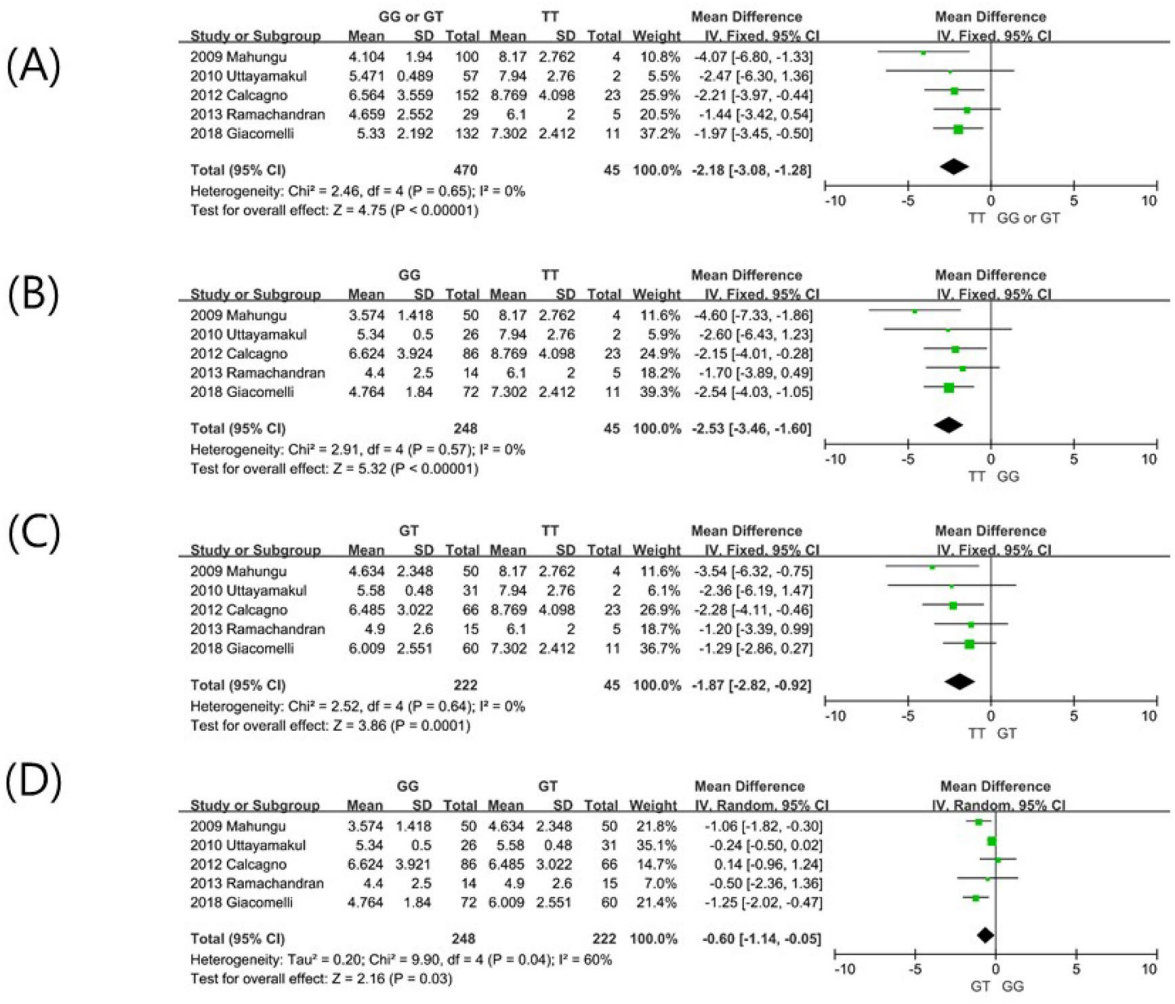
Forest plots demonstrating the association between *CYP2B6* 516 G > T polymorphisms and plasma trough concentration (µg/mL) of nevirapine; (**A**) GG or GT versus TT; (**B**) GG versus TT; (**C**) GT versus TT; (**D**) GG versus GT.

Six studies were evaluated to investigate the association between *CYP2B6* polymorphisms and trough concentrations of NVP, which involved data from 634 patients. The most frequently studied polymorphism was 516 G > T. As one study only compared NVP concentrations between patients with GG genotypes and T allele carriers (GT or TT)^16^, data from 562 patients from the other five studies was included in meta-analysis. NVP concentrations in the *CYP2B6* 516TT genotype was 2.18 µg/mL (95% confidence interval (CI): 1.28–3.08) higher compared with the concentration in the GG or GT genotypes (5.38 versus 7.56 µg/mL; Fig. 2A). There was no heterogeneity observed among studies (I^2^ = 0%). Neither Begg’s test nor Egger’s test showed significant publication bias (Begg’s test: *P* = 1.000; Egger’s test: *P* = 0.580).

In respective comparisons of the three genotypes, every association was found to be significant. The mean difference (MD) was 2.53 µg/mL between TT and GG (95% CI 1.60–3.46; Fig. 2B), 1.87 µg/mL between the TT and GT genotypes (95% CI 0.92–2.82; Fig. 2C), and 0.60 µg/mL between GT and GG (95% CI 0.05–1.14; Fig. 2D).

**Figure 3:**
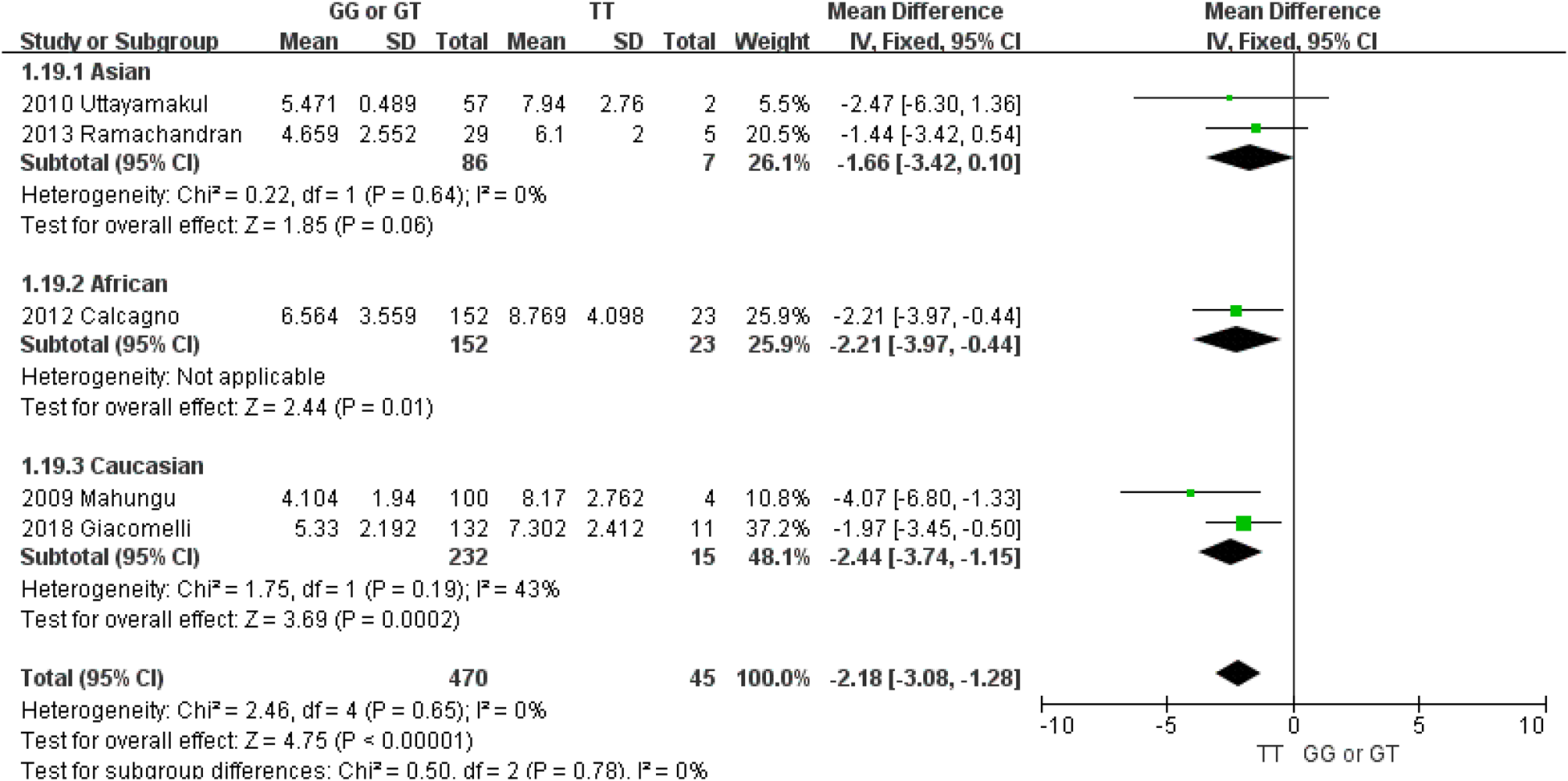
Forest plots of the subgroup analysis demonstrating the lack of association between the *CYP2B6* 516 G > T polymorphism and plasma trough concentration (µg/mL) of nevirapine by ethnicity.

Subgroup analysis by ethnicity was also performed (Fig. 3). The MD between the TT and GG or GT genotypes for Asians, Africans and Caucasians were 1.66 (95% CI − 0.10–3.42), 2.21 (95% CI 0.44–3.97), and 2.44 (95% CI 1.15–3.74), respectively. The subgroup analysis results were similar to that from the entire meta-analysis, and there was no significant difference depending on subgroup (*P* = 0.78).

### Other *CYP2B6* polymorphisms

There were two studies that investigated the polymorphism *CYP2B6* 983 T > C, two studies that investigated 1459 C > T, and one study that investigated 785 A > G (Table 2). For 983 T > C, a study by Calcangno et al*.*^17^ showed that patients with the TC genotype exhibited significantly higher NVP concentrations compared to the TT genotype. Another study showed similar trends^14^; however, statistical significance was not attained. In two studies investigating 1459 C > T^14,16^, wild allele homozygote (CC) carriers displayed higher NVP concentrations than variant allele carriers (CT or TT), although this was also not statistically significant. Regarding 785 A > G, there was only one study^16^ and there was no significant difference between wild allele homozygotes and variant allele carriers.

**Table 2:** Summaries of associations between *CYP2B6* polymorphisms and nevirapine trough concentrations.

First author	983 T > C			1459 C > T			785 A > G		
	TT	TC	*P*	CC	CT or TT	*P*	AA	AG or GG	*P*
Mahungu, 2009	4.17 ± 2.20 (103)	5.806 (1)	0.52	4.34 ± 2.35 (82)	3.62 ± 1.13 (21)	0.15	–	–	–
Gozalo, 2011	–	–	–	5.33 ± 1.91 (47)	4.20 ± 1.87 (11)	0.14	5.00 ± 1.87 (29)	5.02 ± 1.64 (28)	0.85
Calcagno, 2012	6.54 ± 3.49 (153)	8.74 ± 4.24 (19)	0.012	–	–	–	–	–	–

Data were expressed as mean ± SD (number of patients).

## Discussion

This is the first meta-analysis evaluating the influence of *CYP2B6* gene polymorphisms on NVP trough concentrations. HIV–infected patients with the *CYP2B6* 516TT genotype showed higher NVP concentrations than did GG or GT genotypes. Notable statistical significance was also attained in each of the three respective comparisons of genotypes. There was no difference between subgroups by ethnicity.

CYP2B6 is a member of the cytochrome P450 family, constituting approximately 2–10% of the total hepatic CYP content^21^ with significant inter-variability^22^. The difference in CYP2B6 mRNA and protein expression between individuals has been observed to be up to 250-fold and 100-fold, respectively^22,23^. Such variability is possibly due to effects of genetic polymorphisms, with one of the most frequently studied polymorphisms being 516 G > T. *CYP2B6* 516 G > T, one of the two linked nonsynonymous polymorphisms in the *6 allele, is the causal mutation finally leading to reduced function. This results not only in an amino acid change but also in severely reduced levels of functional full-length mRNA transcript, protein, and activity^24^.

Several studies have investigated the association between 516 G > T and drug concentrations. According to these studies, 516 G > T polymorphism was associated with concentrations of drugs that are metabolized by CYP2B6 (e.g., methadone, propofol)^25–27^. In addition, as CYP2B6 is the major enzyme involved in the metabolism of efavirenz (EFV), another NNRTI, its polymorphism was shown to be related to EFV drug disposition. A recent meta-analysis noted that patients with the TT genotype exhibited significantly higher plasma concentrations of EFV compared with those that were homozygous for the G allele (MD: 2.98; 95% CI 2.19–3.76)^28^. Thus, the results of this study that suggest an association between 516 G > T and NVP concentrations are in line with previous research.

There was no significant ethnicity-dependent difference in this study population. Patients with the TT genotype had higher NVP concentrations regardless of the ethnic background. This was consistent with the result from the aforementioned meta-analysis of EFV^28^, which reported that the higher EFV concentration in patients with the TT genotype was found within all ethnicity groups; overall EFV concentration in the TT genotype carriers was comparable between different ethnic groups.

Higher plasma concentrations of NVP is one of the important issues regarding safety. In a clinical test conducted in China, hepatotoxicity was significantly associated with median NVP trough concentrations in male patients (8.20 vs. 5.48 µg/mL, *P* = 0.015), along with hepatitis C virus co-infection (*P* = 0.039)^6^. Gonzalez de Requena et al. also showed that among patients with chronic hepatitis C coinfection, NVP concentrations > 6 µg/mL were associated with a 92% risk of liver toxicity^29^. A multivariate linear regression analysis also revealed that high trough concentrations was an independent predictor for elevated liver enzyme levels^30^. Moreover, several studies reported that the *CYP2B6* 516 G > T polymorphism was associated with adverse events of NVP. According to Yuan et al*.*^31^, cutaneous adverse events were associated with *CYP2B6* 516 T (OR: 1.66; 95% CI 1.29–2.15). Ciccacci et al*.*^32^ also suggested associations between *CYP2B6* polymorphisms (516 G > T and 983 T > C) and SJS/TEN susceptibility. Furthermore, another meta-analysis confirmed that patients with the GT and TT genotypes of 516 G > T displayed a significantly increased risk of EFV-induced side effects compared to those with the GG genotype, possibly due to the slow EFV clearance^33^.

In the selection process for AIDS treatment regimens, toxicity and long-term efficacy are factors of priority. Individual genetic variability may also be related to pharmacodynamics and pharmacokinetics of drugs, thus suggesting a need to develop therapy strategies tailor-made for different patients of varying genetic profiles. In developing countries, where HIV treatment options are limited, it may be especially beneficial to identify individual patient characteristics, including genetic polymorphisms. The data obtained in the present meta-analysis suggest that genotypes of *CYP2B6* may be a useful factor in the identification of HIV-infected patients at risk of higher NVP concentrations.

This meta-analysis has some limitations that should be considered when interpreting the results. First, the limited number of studies may lead to low statistical power in detecting differences or heterogeneity. However, according to Herbison et al*.,* meta-analyses with as few as four or five studies could produce robust results that are consistent with long-term results^34^. Second, some potential factors which could be associated with NVP (e.g., liver functions and concomitant drugs) could not be adjusted due to lack of information from individual studies. Third, the NVP dosing regimen was not identical; we included studies with patients that received either 400 mg once daily or 200 mg twice daily. Despite these shortcomings, this is the first systematic review and meta-analysis to evaluate the association between *CYP2B6* polymorphisms and NVP concentrations. By combining the results of several studies, this current study suggests that *CYP2B6* polymorphisms, especially 516 G > T, may be associated with NVP trough concentrations in a significant way. Therefore, based on our findings, *CYP2B6* genotyping may be useful in predicting response to NVP, although these results must be confirmed in studies on larger populations regarding pharmacodynamics.

## Methods and materials

### Literature search strategy and inclusion criteria

This meta-analysis was conducted according to the checklist outlined in the Preferred Reporting Items for Systematic Reviews and Meta-Analyses (PRISMA)^35^. Two reviewers independently searched published studies, which published until April 2020. An extensive search of electronic databases (PubMed, Web of Science, and EMBASE) was performed using the following search terms: (nevirapine OR NVP OR BI-RG-587 OR viramun*) AND (2B6 OR CYP2B6 OR Cytochrome P450 2B6 OR Cytochrome p‐4502B6 OR CYP2B6* OR P4502B6). There was no limitation on language.

Studies were included if (1) the studies evaluated the relationship of *CYP2B6* genotypes with plasma trough concentrations of NVP (µg/mL) among HIV patients; (2) the patients received full-dose NVP (either 400 mg once daily or 200 mg twice daily) for at least 14 days or achieved steady-state conditions prior to pharmacokinetic sampling; and (3) the NVP concentration in plasma at 12 h after dosing (12 ± 2 h) were available and described separately according to different genotypes. Studies were excluded if they were (1) editorials, notes, abstracts, reviews, comments, letters, news, or editorials; (2) in vitro or in vivo studies; (3) unable to extract the data; (4) studies on children. In case of overlapping data, only the most recent and comprehensive data were included in the meta-analysis.

### Data extraction and study quality assessment

Two reviewers independently extracted data, and discrepancies were resolved by consensus. Extracted data included the following information: name of first author, publication year, number of patients, mean age, percentage of males, ethnicity, studied alleles, and plasma trough concentrations of NVP.

Articles were assessed by two investigators based on the JBI Critical Appraisal Checklist for quality assessment. Quality was quantified by assigning scores ranging from 0 to 1 point/criteria. One point was assigned if the item was expressed in the study, and zero points were given if the item was not expressed or if it was unclear.

### Statistical analysis

Meta-analysis was performed using Review Manager (version 5.3; The Cochrane Collaboration, Copenhagen, Denmark). The MD and 95% CIs were used to identify the relationship between the existence of *CYP2B6* polymorphisms and plasma trough concentrations of NVP. For studies that only reported medians and interquartile ranges, we retrieved mean and variance values from authors of original reports or used appropriate formulas to calculate mean and variance, making no assumption on the distribution of the underlying data^36^. The heterogeneity across studies was estimated by way of a chi-square test and an I^2^ statistic. An I^2^ value of ≥ 50% was considered to indicate significant heterogeneity. The choice of the proper effect model was based on the analysis results: the fixed effect model was used if I^2^ < 50% and the random effect model was used if I^2^ ≥ 50%^37^. Subgroup analysis was performed according to ethnicity. Both Begg’s rank correlation test and Egger’s regression test of the funnel plot were also conducted using R Studio software (version 3.6.0; R Foundation for Statistical Computing, Vienna, Austria) to detect publication bias^38,39^. A *P*-value < 0.05 was considered statistically significant.
